# Designing a tobacco counter-marketing campaign for African American youth

**DOI:** 10.1186/1617-9625-4-7

**Published:** 2008-08-26

**Authors:** Doris M Johnson, Lauren A Wine, Sharon Zack, Eric Zimmer, Judy H Wang, Patricia A Weitzel-O'Neill, Vickie Claflin, Kenneth P Tercyak

**Affiliations:** 1 Department of Psychology & Counseling, University of the District of Columbia, Washington, DC, USA; 2 Cancer Control Program, Lombardi Comprehensive Cancer Center, Georgetown University Medical Center, Washington, DC, USA; 3 Danya International, Inc., Silver Spring, Maryland, USA; 4 Communication, Culture, & Technology Program, Georgetown University, Washington, DC, USA; 5 Catholic Schools Office, Archdiocese of Washington, Washington, DC, USA

## Abstract

The objectives of this qualitative study were to: a) identify common marketing themes and tactics used by the tobacco industry to entice African Americans (AA's) and youth to initiate and maintain smoking behavior, especially smoking mentholated brands of cigarettes, and b) determine AA youths' knowledge, attitudes, intentions, and beliefs about smoking and the tobacco industry. Together, these activities could aid in the development of effective tobacco counter-marketing campaigns for AA youth. Using publicly available tobacco industry documents, computerized searches using standardized keywords were run and results were cataloged and analyzed thematically. Subsequently, 5 focus groups were conducted with n = 28 AA middle school-aged youth. Results suggest that the tobacco industry consistently recruited new AA smokers through a variety of means, including social and behavioral marketing studies and targeted media and promotional campaigns in predominantly AA, urban, and low income areas. AA youth interviewed in this study were largely unaware of these tactics, and reacted negatively against the industry upon learning of them. Youth tended to externalize control over tobacco, especially within the AA community. In designing a counter-marketing campaign for this population, partnering knowledge of tobacco industry practices with youth needs and community resources will likely increase their effectiveness.

## Introduction

In the United States and in many other parts of the world, tobacco use is the leading preventable cause of lung cancer and other chronic diseases of adulthood, and cigarettes are the most commonly used form of tobacco [1]. Approximately 22% of adults in the United States are current cigarette smokers, with prevalence rates of 24% among males, 19% among females, 23% among Caucasians, and 22% among African Americans (AAs) [2]. AA males suffer disproportionately from the morbidity and mortality of smoking as they have the highest rates of lung cancer and lung cancer-related deaths than any other racial or ethnic group [3]. In addition to sex and race, socioeconomic status is a leading smoking risk factor as well. Estimates are highest for adults with General Education Development diplomas (44%), those who did not complete high school (34%), and individuals who live below the poverty level (31%) [2].

For these and other reasons, the District of Columbia (DC) is among the areas of the United States hardest hit by smoking and cancer. Approximately 60% of residents are AA [4], 22% have not graduated from high school, and 20% live below the poverty level [4]. Further, 1:4 AA adults in DC currently smoke [2], and the lung cancer incidence rate among AA men and women in DC is 84:100,000 [5].

Much of the origin of adults' lung cancer risk begins in childhood, where beliefs, attitudes, intentions, and behaviors regarding cigarette smoking are first formed and track into adulthood [6]. Data from the 2002 National Youth Tobacco Survey reveal that in middle school (grades 6–8) 33% of students have ever smoked (37% among AAs) [7]. By the time children reach high school, 57% have ever smoked (57% among AAs) [7]. Mentholated cigarettes are overwhelmingly preferred by AA youth, with 64% of AA middle school smokers and 79% of AA high school smokers choosing mentholated brands [7]. Regarding AA middle schools students' beliefs about cigarette smoking, 62% believe smokers may have more friends and 25% believe smoking makes them look cool [7]. In terms of their intentions to smoke, 28% intend to smoke this year, 32% intend to smoke within the next 5 years, and 30% intend to smoke a cigarette if offered by their friends [7]. If these trends continue, >7,000 youth alive today in DC are expected to die from smoking [2].

To address the growing public health crisis generated from the use of tobacco products in the United States, countless health education campaigns have been launched to deter youth from smoking. Among the more effective campaigns are those that rely on counter-marketing approaches [8-13]. As noted by the CDC, counter-marketing consists of attempts to: "counter pro-tobacco influences and increase pro-health messages and influences throughout a state, region, or community" [14]. Counter-marketing campaigns accomplish their goals by arguing against, contradicting, or offsetting, the promotional activities of the tobacco industry, such as annual industry spending (which tops $9.57 B overall, $170.2 M on advertising and promotions, and $949,000 on Internet advertising) for magazine and other ads for youth [14]. Among the characteristics of promising tobacco counter-marketing campaigns are those utilizing integrated components to deliver messages (e.g., public service announcements, media literacy training, and classroom discussion), integrating the campaign into other tobacco education, counseling, cessation, and policy efforts, ensuring the campaign's cultural competence, and making strategic decisions about the campaign's target audience, creative products, and delivery [14].

The American Legacy Foundation's "truth" national media campaign is one example of this approach. Truth seeks to counter pro-tobacco messages delivered via the tobacco industry by alerting youth to the industry's marketing practices that glamorize smoking but without mentioning addiction or health consequences. Youth exposed to the campaign through broadcast media advertising hold more negative beliefs about tobacco industry practices and more negative attitudes about the industry itself [15]. Additionally, these negative beliefs about the tobacco industry are, in turn, linked to lower receptivity to pro-tobacco advertising and less progression in smoking intentions and behaviors [15]. However, observed effects are not completely even with respect to age and race. Younger children (i.e., middle school students) and AA's were differentially impact by the campaign, with younger children less affected than older children and AA's more affected than other groups. This suggests that the highly promising counter-marketing approach advocated by the CDC for state-wide tobacco control initiatives [14] may effectively reach a target audience of AA's, though the developmental level of these youth will be important [16].

Focus group research (e.g., small-group research conducted with a few members sampled from a larger target population who openly discuss a particular subject or area) to assess the perceptions of the use of tobacco products among AA high school students and adults is available. For example, Malone and colleagues (2001) conducted focus groups with 14–18 year-old AA's and found that perceptions of tobacco risks are large determinants of usage patterns [17]. A later study by Crawford and colleagues (2002) conducted 129 focus groups (36 of which comprised AA teenagers) to explore adolescents' response to tobacco control policy [18]. The results of the study suggested that teenagers' input most likely increases the effectiveness of control efforts. Yerger and colleagues (2005) presented internal tobacco industry documents to AA 18–34 year-old adult focus group members [19]. The purpose of that study was to identify potential targets of tobacco control intervention. Those results indicated that such documents are likely to be useful in tobacco efforts involving counter-marketing. Other research has focused on attempts by adults and teens to stop smoking, and find that more education and counseling in this area is warranted, especially among AAs [20,21]. However, scant empirical research has focused on AA middle school students exclusively, especially investigations involving qualitative methods and those intended to generate hypotheses for further research.

Qualitative methods are well-suited toward this enterprise, as they gather nuanced information about perceived and voiced experiences people may hold. According to a scientific expert panel convened by the National Institutes of Health at the United States Department of Health and Human Services: "Qualitative methods are often employed in unstudied or understudied areas" (1999) [22]. That panel went on to state that when qualitative work in an area of science has shown promise, additional qualitative research in that area may be warranted. When applied to the problem of tobacco use among AAs – and AA youth more specifically – such methods could be useful in furthering the understanding of how to best prevent and control tobacco use in this population.

Shervington (1994) conducted a qualitative investigation of cigarette smoking among AA women and reported that cultural-competence should be a core component of smoking cessation work with this population [23]. Another qualitative report by Gittelsohn and colleagues (2001) focusing on cigarette smoking among AA youth was highly informative with respect to social context risk (e.g., presence of parental smoking, absence of strong anti-smoking policies at school) and protective (e.g., desire not to disrespect parents) mechanisms that affect AA smoking uptake, with notable implications for intervention [24]. Finally, in reporting on the results of a smoking cessation trial conducted with AA adults, Woods and colleagues (2002) used qualitative methods to better understand why AAs may be prone to low enrollment into clinical trials of smoking cessation (e.g., poor transportation, difficulty obtaining time off from work) [25]. These studies provide key examples of where qualitative research has been used to inform tobacco prevention and control among AAs. The current paper applies these methods to AA middle school students, with the hope of gaining important insights into promising counter-marketing strategies for this group [26].

In light of these issues, the goal of this research was to initiate the development of a tobacco counter-marketing campaign for AA middle school students in Washington, DC. This work was accomplished in two phases. Phase I consisted of the retrieval and review of over 200 internal tobacco industry documents made publicly available following the Master Settlement Agreement of 1998 between the United States Attorneys General and the tobacco industry [27]. The aim of this phase was to identify common marketing themes and tactics by providing specific examples of local, state, and national marketing of cigarettes and other tobacco products to AAs and youth. Phase II of the study consisted of focus groups conducted with AA middle school students attending school in Washington, DC. The aim of this phase was to determine participants' knowledge, attitudes, intentions, and beliefs about smoking and the tobacco industry. When joined together, we expect the results of these two phases could significantly aid in the development of effective tobacco counter-marketing campaigns for AA youth.

## Methods

### Phase I

#### Online document review

Consistent with the methods outlined in Cummings and colleagues (2002) [28], the research team conducted a comprehensive review of online tobacco industry documents available at Tobacco Documents Online (TDO; ) [29]. TDO is an Internet-accessible and searchable repository of high-quality, electronic images of industry documents, with optical character recognition capability, and the ability to collect and annotate documents.

In preparation for using the TDO system, a trainer from the Roswell Park Cancer Institute developed and delivered a two-day workshop for the study investigators. The workshop presented effective strategies for online searching of internal tobacco industry documents and strategies used by Cummings and colleagues (2002) [28]. Each of the major United States tobacco companies was considered, with particular emphasis placed on brands targeted to and preferred by AAs.

Initial search terms were guided by the scientific literature and further refined as terms were identified within documents themselves. A partial list of search terms is provided in Table 1 (adapted from Cummings et al., 2002) [28]. After identifying a potentially-relevant document, it was cataloged into an electronic database. Cataloging included the document's TDO-assigned date, hypertext link, title, and author, key words used to generate the document, company, brief abstract, and date of retrieval. Following the initial cataloging of the documents, each document was then reviewed during a research team meeting and determined to be relevant or not for the study's purpose based on the research protocol. Our protocol specified that the criteria for determining a document's relevance was based upon one or more of the following attributes: (1) novelty, (2) relationship to youth, and/or (3) relationship to AAs. In all, 144 out of the 236 documents retrieved (61%) were retained and analyzed qualitatively.

**Table 1:** Search terms

Addiction	Helping youth decide	Promotions
Advertising	High school	Sex
African American	Initiation	Smooth
B1G1F: buy 1 get 1 free	Inner-city	Starters
Black	Low socioeconomic status	Students
Brand, brand loyalty	(SES)	Surveys
Campaigns	Low income	Switching brands
Children	Lung	Teens
Cigarette machines	Market	TGMP: target group
College	Menthol	Meeting place
Colored	Minority	Underage
Education	MTV	Urban
Filter	Negro	We card
Free samples	Peer pressure	YAM: young adult male
FUBYAS: first usual brand young adult smoker or first unbranded young adult smoker	Peer, peer brand	YAS: young adult smoker
Brand, young adult smoker	Point of sale	Young
Gangster	Poor	Young adult
Generation X	POP: point of purchase	Youth
Give aways	Poverty	
Health	Project Scum, P.O.W.	

### Phase II

#### Focus groups

A total of five focus groups were conducted within four different parochial elementary schools in Washington, DC. DC parochial schools serve over 4,400 students in 20+ schools in kindergarten through eighth grade and approximately 60% of their student population is AA. The four participating schools were selected based on the criteria of schools serving high proportions of low-income, underprivileged, AA youth.

Focus group participants were recruited through two waves of direct mailings sent from each school's principal to the child's parent or legal guardian. The mailing included a cover letter from the school principal explaining the purpose of the study, along with an adult informed consent form, a child informed assent form, a family demographic form, and a postage-paid envelope to facilitate the return of signed consent forms. Active (written) parental consent and child assent were sought and required prior to participation. Each participant was provided with a snack and beverage during the group.

In the first wave of study mailings, a total of 225 invitation letters were sent. Of those, a total of 22 signed consent forms were returned. In the second wave of mailings (approximately 3 weeks later), a total of 189 study invitation letters were resent to non-responding parents or legal guardians with valid home address information and 8 consents were obtained. Combined, this represents a study consent rate of approximately 13% (30/225).

Focus groups were scheduled in advance and occurred during participants' free periods. Only participants attending school on the day of a focus group were permitted to participate; two participants were excluded from participation due to this reason. All groups occurred on school property, usually in an available classroom. Two professional moderators led the groups. Both moderators were female and the senior moderator was AA. Focus groups were audio recorded and detailed observational notes were taken throughout the sessions; each session was conducted until a saturation point (point of diminishing return) had been reached and typically lasted 60 minutes or less.

Following the administration of a brief demographic questionnaire, focus group participants were led through a series of planned, tobacco-related discussions of their knowledge, attitudes, intentions, and beliefs related to cigarette smoking, tobacco industry marketing practices, friends and family members' smoking habits, and advertising and persuasive communications. Focus group questions were derived in advance and contained in a written moderator's guide. However, group dynamics shaped the process and content of each discussion. All youth were encouraged to participate with one another and with the focus group moderators.

At the conclusion of all focus groups, audio recordings and observational field notes from each session were transcribed and analyzed by members of the research team (D.M.J., L.A.W., S.Z., E.Z., J.H.W., K.P.T.) using an iterative approach based upon Grounded Theory [30]. This theory essentially refers to an iterative, inductive reasoning process that has increasingly been utilized in the social and behavioral sciences – whereby a conceptual model or framework emerges over time from data and evidence that have been systematically gathered and obtained. In its implementation, Grounded Theory method is akin to a logic or problem-solving activity focused on deepening one's understanding of behavior from the perspective of individuals or groups of individuals of interest. Common sources of data include interviews and observations analyzed using codes and sampling procedures. Consistent with Grounded Theory, the net result of this activity is a fuller understanding of the nature of a given phenomena, and the ability to better characterize or intervene upon it in the future.

In this case, each focus group's transcript was read independently by each research team member. Next, all research team members met in conference to consecutively review each transcript in detail. Third, team members' worked together to categorize each transcript's content according to inductively-developed thematic codes relating to specific quotations offered by participants and relevant text. All codes and text were then reviewed together by the research team one final time to compare and contrast each one and resolve any remaining discrepancies by consensus.

Study procedures were approved by the Institutional Review Boards of the University of the District of Columbia and Georgetown University Medical Center and by each school.

## Results

### Phase I

The study team qualitatively analyzed database records to identify common marketing themes and tactics employed by the tobacco companies. In all, five key themes were identified pertaining to AAs and youth within and outside of our geographic area. These five themes, their descriptors, and illustrative quotations are provided below:

#### 1. Recruiting new smokers via industry studies of youth culture

The online documents suggested that tobacco companies systematically engaged in market research to generate data on AA demographic trends, smoking patterns, and attitudes towards smoking. Tobacco companies sought information and support on specific strategies that would be effective in enticing AA adolescents to begin smoking early and continue into adulthood. Internal tobacco company memos revealed tobacco executives' awareness of the declining critical mass of potential new smokers and the need to use effective advertising strategies to increase market share.

Recent advertising on Newport geared towards the younger generation with the Negro male wearing the blue dashiki has received a tremendous amount of praise from the consumer. [31]

As we know from the literature, as well as from our own experience with Marlboro, the best way to get a foothold in a market is to catch the users of a product when they are young, give them what they want, and establish that brand as the brand of choice among the trendsetters. It then becomes the brand of choice for others in that and subsequent age cohorts, and all carry it along to adulthood, either in its original form or in the form, of a line extension. In the case of Marlboro the trend-setters were college bound upward mobile white males. In the case of Newport they appear to be young upward mobile blacks of both sexes, although it is also attracting a frighteningly large proportion of young white collar whites as well. [32]

#### 2. Understanding regional and other differences affecting smoking uptake, maintenance, and brand loyalty via industry studies of African American culture

Tobacco companies' data indicate that AAs are a leading market segment that purchases and consumes menthol cigarettes. Concerns over menthol cigarette market share were expressed in direct relation to data indicating declining market share, shifting brand loyalty, and brand switching among AAs.

The basic objectives of the study were: to determine the relevance of the Marlboro masculinity concept to urban black cigarette smokers. [33]

In order to get a foothold in this young black menthol market we have to offer them a cigarette that they want and what they want appears be a high delivery cigarette. Therefore I remain convinced that in order to crack this market we will have to have a free-standing menthol brand with about 16 mg delivery (perhaps simply a repackaged Marlboro Menthol) with a short, easily pronounced name, marketed to (but not specifically for) blacks. [32]

#### 3. Investing in the African American community, ethnic, and cultural events to enhance the industry's image

Tobacco companies successfully penetrated AA cultural activities such as ethnic festivals, jazz music events, scholarship programs, and product giveaways. Successful efforts also led to partnerships with prominent AA community groups, such as the National Association for the Advancement of Colored People, National Urban League, and National Coalition of 100 Black Women. The increased presence and improved relationships upgraded the industry's status within the community.

KOOL is to develop programs which ingratiate themselves with the Black community. These programs are to show the makers of KOOL as a community citizen, be backfire- proof and pave the way for supporting the brand. [34]

Philip Morris staff has reported that the following groups have or will submit statements in support of our position: NAACP, National Urban League, National Association of Black County Officials, National Coalition of 100 Black Women, National Black Police Association, Uptown (NY) Chamber of Commerce, National Minority Supplier Development Council, National Minority Business Council, National Association of Minority Contractors, Asian-Pacific American Chamber of Commerce, West Coast Black Publishers Association, Leadership Conference on Civil Rights, U.S. Hispanic Chamber of Commerce, and the Georgia Association of Black Elected Officials. [35]

#### 4. Targeting low income smokers and youth in urban areas

Billboard advertising of cigarettes was a vehicle primarily used in urban areas with large minority communities and low socioeconomic status.

Outdoor $30 MM [$30 million]. Most efficient media form. Primary media form to reach down scale blacks and young adult males. [36]

Whereas our company's Newport is the number one brand among young African-American males and females; – 80% of Loews ad dollars go for Newport. From July-September, 1986 $4.7 million of the $6.5 million spent advertising went into billboards. Over 50% of billboard advertisements in low-income minority communities feature alcohol and tobacco. 70% of Newport advertising goes into billboards. [37]

#### 5. Targeting African Americans through geography and urbanicity

Tobacco companies concluded that the young urban AA did not fit the profile of the traditional smoker. The young urban AA was considered a particular subset of the smoking population that responded to specific environmental factors.

The NEWPORT brand is appealing to a different "mind-set", predominantly among males, in the Black inner city. [38]

But the 'inner-city' Black smoker is not part of the 'traditional' market. They have shown themselves basically impervious to print media exposure, outdoor is limited and most importantly, they have high susceptibility to peer group influence. [38]

### Phase II

Demographic characteristics of focus group participants are presented in Table 2. Schools ranged in size from 178 to 214 students across pre-kindergarten through eighth grade, with between 96% and 100% AA students per school. Focus group participants ranged in age from 10–14 years.

**Table 2:** Focus group participant characteristics

		**Group**				
	**Total**	**1**	**2**	**3**	**4**	**5**

N	28	7	8	4	6	3
n Grade 6	9	2	3	4	0	0
n Grade 7	13	3	4	0	3	3
n Grade 8	6	2	1	0	3	0
M Age (years)	12.3	12.4	12.1	11.8	12.8	12.0
n Male	17	3	6	1	4	3
n Female	11	4	2	3	2	0
n Ever smoked (yes)	4	2	0	1	1	0

Note. Groups 3 and 4 conducted at same school.

Focus group discussions were organized around five major themes, as directed by the moderator's guide: (1) perceived behavioral epidemiology of tobacco use, (2) awareness of tobacco industry marketing, (3) attitudes toward tobacco use, (4) influences on intentions to use tobacco, and (5) educational interventions to counteract industry marketing.

Perceived behavioral epidemiology describes what participants know about cigarettes and their impact, with whom adolescents smoke, and where, when, and why they smoke. Awareness of tobacco industry marketing includes knowledge that AA youth are being targeted by tobacco industry marketing and feelings about this fact. Attitudes toward tobacco use refers to opinions and feelings about smoking among youth. Influences on intentions to use tobacco describe commercial and social influences on directed use of cigarettes. Finally, we solicited participants' ideas about creating a culturally-competent intervention program to counteract industry marketing to AA youth. These five themes, their descriptors, and illustrative quotations are also provided below:

#### 1. Perceived behavioral epidemiology

The majority of participants recognized the negative impact of tobacco use and were favorably inclined toward anti-smoking initiatives. Participants referred to cigarettes by several terms, including "devil", "jack", "blunt", "joe", "tobacco chew", and "pack of smokes". Some participants identified cigarettes by attending to their health-compromising qualities (i.e., "cancer sticks", "death in days"). Many acknowledged that smoking increased risk for developing lung cancer and caused long-term consequences.

It can give you lung cancer and it can, um, it can, um, mess up your brain.

Female, Focus Group (FG) 2

It can affect your mind...in, like, like, physical, you can have physical changes and, uh, you can have...changes

Male, FG 5

By contrast, at least one participant did not agree that smoking is threatening to health.

I don't believe [inaudible] that health, I mean, smoking will kill you, well, I mean, especially tobacco, I mean, tobacco isn't really seriously causing you to die, I mean, that's how I feel...because a lot of people, mostly all the people, or all the grown people that I know have been smoking all they lives and nothing has happened to them and they haven't caught a lung disease or, you know, caught, they never got anything, as far as I'm concerned they still do it and so...the people that I know they smoke but nothing really, nothing bad happens.

Female, FG 1

Participants reported that other youth their age smoke in multiple physical locations and settings (e.g., with friends, older high school students, a group of people in the car, at bus and train stations, by stores, on corners and streets, in alleys, at the basketball courts, and in the back of the school building). Few revealed that youth smoking takes place in their home and with family members present.

Sometimes the people that I know, sometimes, they smoke with their brothers or sisters or cousins or something...no, cause you know that, that's your, that's your brother or sister and you know if you smoke with them they won't go tell mommy or whatever because you all family.

Female, FG 1

#### 2. Industry marketing awareness

When asked whether participants were aware that tobacco industry marketing practices have strategically targeted AAs, some expressed beliefs that campaigns were created for the general population, while others recognized targeting tactics.

Well, as I got older, I, started understand it...they just trying to say all this stuff for us to start smoking and knowing that it might, you know, it might be bad for your health or it's not cool or whatever.

Female, FG 1

On the [mass transit system], like, you know how they have the little thing on there, like, on the sides of the buses...yeah, it's like, it's like, it always be a black person on there and they be having, like, different flavors of the cigarettes and stuff.

Female, FG 2

A good number of participants expressed anger toward the tobacco industry's attempts to recruit young AAs to smoke, but they were not surprised to learn of this practice. Many regarded this target marketing as a result or form of racism.

Why target one group when you've got a whole, you got other set of groups in the U.S. and, um, it seems unfair

Male, FG 3

Participants also felt that AA youth were more susceptible to using tobacco than other ethnic groups since they had lower in-school rates and knew less about the negative effects of smoking on health.

I think they target us [AAs] more because of, you know, if, now today, it's more, it's more popularity of teens and adults that don't go to school, they don't have jobs, I mean, for real, they don't have anything to do.

Female, FG 1

When the people look at the African Americans, they's like...they don't know the facts about what's bad for them, bad effects about it, they just know, like, things that be encouraging them, like, videos with people who smoking.

Male, FG 1

Some participants internalized this information and expressed stronger intentions to avoid smoking.

It makes me want to not do it even more.

Male, FG 3

On the contrary, at least one participant claimed that smoking was a personal choice that should be respected. Another participant expressed distress that smokers appeared to have lost self-control and were being exploited by industry marketing.

That makes me feel mad because those people who start to smoke it makes me think they don't have a mind of their own, that they follow other people and what they see on TV.

Female, FG 2

In discussing ways to prevent or control the tobacco industry's attempts to market cigarettes to AA youth, participants focused more heavily on external (rather than internal) sources of behavioral regulation and control. For example, the President of the United States and the government in general were emphasized as the most powerful authorities to regulate tobacco industry marketing. Successful and well-respected members of the AA community were also deemed as influential figures to limit target marketing. Fleetingly, some participants suggested that customers could have a negative impact on the tobacco industry by boycotting tobacco products, protesting, petitioning the government to act, and by raising cigarette prices.

#### 3. Attitudes toward tobacco use

Participants reported that youth smoked for various reasons, including desires to be cool and popular, to appear mature and in control, to fit in with their peer groups, and to alleviate stress.

I think it makes them [smokers] feel like, oh, they uh, they get, they, uh, trying to be cool and stuff and trying to fit in because everybody else smoking.

Female, FG 2

That's what my mother do she, uh, she said she smoke when she stressed.

Female, FG 2

In addition, some participants recognized the downside of smoking and regarded it as being physically unattractive with negative consequences of nicotine withdrawal.

If you smoke long enough, yes, your lips will start turning black and people will start noticing that you are smoking, that's why there a lot of boys out there that's not attractive anymore because their lips turned all black.

Female, FG 1

If you smoke one day, it's like, it mess with you head, so you don't, like, if you smoke one day before the test, its like, you'll not be able, you're not going to be able to concentrate.

Male, FG 1

When asked what they would do if offered a cigarette, most participants responded that they would walk away and found ways to refuse the offering. However, peer pressure interfered with refusal.

I would say no and then if they kept trying to force me I would go get my mother.

Male, FG 2

You shouldn't offer someone my age a cigarette because it's bad

Male, FG 4

Most kids give in to peer pressure because they don't know how to say no.

Male, FG 5

Participants complimented and admired others who refused cigarettes. Although a few participants expressed understanding about why their friends smoke, the majority indicated that smoking was unacceptable and jeopardized friendships.

I wouldn't be able to trust them no more 'cuz, like, when people get [smoke] they start doing crazy stuff.

Male, FG 2

Participants reportedly valued education and knowledge as means to empower AA youth to avoid smoking.

Like why would you waste your life, like, like, some people they may have a good talent, like, they might be able to sing or [inaudible], or draw, or like with the singing, if you smoke, you, like, mess up your chance, because you could end up having cancer, and going to the hospital and mess your whole life up.

Male, FG 4

#### 4. Influences on intentions to use tobacco

On balance, focus group participants were skeptical that exposure to tobacco industry marketing would increase their own intentions to use tobacco, but agreed it might affect initiation among their peers.

It really depends on how the person feel because it, like, for me I think smoking is stupid but say, like, somebody else that's my age might think it's not, so it depends on how they feel.

Female, FG 2

When asked to recall tobacco product marketing campaigns, participants explained how campaigns shaped their perceptions of cigarettes.

They in the, the Kool magazine they always have black people smoking...they were smoking and having fun...just standing up, like laughing.

Male, FG 2

While asked to recall counter-marketing campaigns and anti-smoking campaigns participants recalled that campaigns often imitated talk between family members, incorporated high profile role models (e.g., famous actors), and incorporated creative design elements.

I was watching MTV and they had the little commercial and they, uh the man would open the curtain and then show how many people die off of cigarettes.

Male, FG 2

I remember on one of these commercials they, they were, um, talking about, they had this lady on there, uh, she couldn't speak anything because of, uh, lung cancer. So they had the little machine [inaudible] to do it for her.

Male, FG 4

Participants also pointed out that family, peers, and role models all play important roles in behavioral activation and inhibition of smoking. Familial beliefs in particular were noted to strongly affect smoking intentions.

Some people, um, in my family thinks it's good and it's fun and they like playing with them [cigarettes]...some people in my family think it's fun...yeah, so they be joking around with them.

Female, FG 5

Sometimes my mother tell me not to do something, like, go, whatever, do this, and I do it just to see what it's like, but if it's stuff, if it's stuff like health related, like, she tell me don't smoke or don't do this I won't do it because I know it's beneficial to me not to do it.

Female, FG 3

#### 5. Education and counter-marketing

When asked how to assist AA youth to refuse and stop smoking, participants proposed several concepts such as testimonials by students and celebrities, concerts performed by signers or local talent, and the use of posters or other small media.

Get Michael Jordan to come on TV saying something...because everybody look up to Michael Jordan....and put it on, like a local channel or something.

Male, FG 5

Sponsor concerts and fundraisers...let all people our age know that it's not good to smoke and if you are smoking you need to stop...oh, Avra Reed 'cuz she's, she's from DC...I would want to get people in the area because, um, that's who we look up to.

Male, FG 3

We'd have like signs that show how lungs look after people start smoking.

Male, FG 2

On a t-shirt you could have, um, you could have advertisement of smoking is fake you need to see the real picture and then you could show, yeah, like what she say, you could show before and after picture.

Female, FG 2

Other useful forms of communication included websites, telephone counseling, DVD and digital media, or books for young AAs to learn about the negative impact of smoking on health and well-being.

Making a website on not, stop smoking...it would have people with little bubble names of information on how to stop, what can happen to you when you smoke and little flashes, like, the little, the things that pop up, the things that pop...pop up and stay stop smoking, gives you lung cancer.

Male, FG 3

And then I would have a phone line, a phone line for those who smoke and don't and they feel like their friends do and they feel like they have peer pressure over it, they could call and, like, people could be on the lines talking to them and helping them 'cuz they may not want to tell their parents but at least they'll have somebody their own age to tell them so that they, um, they'll have somebody to bond with.

Female, FG 2

## Discussion

The data from this study provide further evidence that the tobacco industry has aggressively targeted AA communities and youth in order to promote the use of cigarettes by these groups. The findings complement those of Cummings and colleagues (2002) and Balbach and colleagues (2003) who reached similar conclusions using similar methods and approaches to those implemented here [28,39]. Namely, the review of internal tobacco industry documents made publicly available as a result of the Master Settlement Agreement of 1998.

One may infer from the internal tobacco industry documents reviewed as part of this research that AA youth in urban areas such as Washington, DC are a particularly vulnerable population targeted by cigarette manufacturers. This may, in part, be due to the demographic characteristics of urban DC, including its high concentration of AA residents, low high school graduation rates, and high poverty level – all of which are positively associated with smoking, including mentholated brands which are the preferred brand of AA's [2,40]. Over time, the positive impact of tobacco marketing and cigarette promotions on AA youth smoking status [41,42], and the negative impact of smoking on the health and well-being of AA youth, are pronounced [43].

One of the goals of this line of research is to better inform the tobacco control community and the public at large about industry strategies in an effort to better manage and change the social climate surrounding cigarettes and to prevent AA youth from smoking. The findings reported herein on five themes used by the industry to promote smoking among AA youth address this goal directly. An additional purpose of identifying such themes is to incorporate them into tobacco counter-marketing campaign efforts. This is done in the hope that being more informed about such tactics enables youth to more accurately identify the intent of pro-tobacco messages (i.e., to initiate and maintain smoking behavior). With this realization, favorable attitudes toward the tobacco industry are expected to erode, receptivity to tobacco industry marketing may decrease, and the chances of starting or continuing to smoke are lessened [15].

Smoking prevention and effective tobacco counter-marketing take on even greater significance when the focus of interest is AA youth. When paired with an understanding of what AA youth in urban areas know, think, and feel about smoking it may further guide and inform the development of culturally-competent anti-smoking campaigns for this special population.

The American Legacy Foundation and other groups employing counter-marketing strategies have been highly successful in doing so – largely by exposing youth to the internal workings of the tobacco industry and monitoring youths' responses to these facts [14,15]. Similarly, in their qualitative study with AA adults exposed to tobacco industry documents, Yerger and colleagues (2005) reported: "...the responses from the focus group participants suggest that viewing these documents had a 'consciousness-raising' effect, disrupting and problematizing their previous understandings about tobacco use and prompting reflective dialogue about the role of the industry in their communities." [19]. Whether or not these same effects would be readily achievable or have a similar impact among AA youth remains to be seen. Nevertheless, data presented herein are encouraging.

Toward that end, our focus group results suggest AA youth in urban areas like Washington, DC have significant exposure to cigarette smoking. Youth have been introduced to smoking through various social contexts, including their interactions with family and friends, the media, tobacco industry marketing, other promotional activities sponsored by the tobacco industry, and through social activities common to urban community settings. Social context and social factors exert considerable influences over youth behavior, including smoking uptake [44]. Thus, all youth must be considered somewhat at risk for smoking initiation and progression over time.

The industry's association of smoking with "coolness" has long shaped social perceptions of the benefits of smoking, including smoking mentholated cigarettes [45]. Research suggests that beginning at an early age, children associate smoking with looking and feeling cool, social acceptance, stress reduction [46]. These perceptions are directly shaped by attractive models used in tobacco advertisements and other ad features [47].

Results from our focus group discussions further indicate that AA youth, in general, are not aware that the tobacco industry has tried to attract them to smoking. Few participants were able to successfully recall target marketing strategies after being prompted to do so. On the one hand, awareness of being a target of the tobacco industry generated negative feelings about this practice, and AA youth reacted by expressing feelings of anger and racism. On the other hand, participants also stated their beliefs that AA youth were easy targets of the tobacco industry because they were less likely to stay in school. Thus, ways to prevent AA youth in urban areas from smoking may include promoting school attendance and providing school-based intervention. Youth's own ideas about how to better prevent tobacco use with the AA community emphasized external control mechanisms (i.e., through the executive and legislative branches of United States government) and deemphasized internal controls. This, too, is important to note when designing effective tobacco counter-marketing campaigns for AA youth, and suggests that attempts to boost principles of self-control are warranted. Though most youth are educated about the negative consequences of smoking, strengthening their inner values against smoking and reducing their tolerance for smoking might be key. In prevention, knowledge is a necessary but insufficient determinant of change in youth [48]. Ultimately, successfully inoculating them against tobacco industry marketing practices and other social influences will also require enhancing tobacco rejection skills.

Beyond public policy, focus group participants also noted the influences of family, friends, and mass media on control over smoking. Possible sources of social support for not smoking included parents, older siblings, peers, and opinion leaders. With regard to opinion leaders, they were typically successful community members or popular culture icons who might be able to motivate youth to embrace anti-smoking attitudes and values and to resist pro-smoking persuasive communications. These and other influential figures in the AA community could play a role in counter-marketing campaigns. I t is noteworthy that many of these same strategies have also been employed by the tobacco industry in luring youth into initiating and maintaining smoking [49,50]. Some youth also suggested that peers who speak out against smoking might be beneficial, as might the use of small media (i.e., posters and t-shirts) to deliver messages that industry-sponsored tobacco advertisements are dishonest. Other modes of health communication included technology, such as Internet sites and telephone counseling.

In sum, Phase I of this research detailed common marketing themes and tactics used by the tobacco industry to target AAs and youth into smoking. These themes could serve as a basis upon which to build and deliver counter-marketing messages to AA youth. In Phase II, qualitative methods were again used to gather nuanced information about AA youths' awareness about smoking and tobacco industry practices – in the hope that this information would also inform counter-marketing intervention research. For example, an AA youth counter-marketing campaign might seek to expose the great lengths the tobacco industry will go to in order to learn about AA youth through demographic and market research. In doing so, it is likely that AA youth will respond negatively to this information and reject tobacco. To increase the likelihood that desired effects are achieved, the results of Phase II suggest that interventions not assume that AA youth are especially well-informed about (or aware of) the true harms of smoking, the addictiveness of nicotine, and that they are being targeted by industry marketing. As part of comprehensive tobacco control efforts, these interventions may also benefit from recognizing the stress-reducing role that cigarettes are believed to play and that, though well-intentioned, AA youth are susceptible to smoking – especially if they lack refusal skills. Education and counter-marketing campaigns delivered within this special population might also benefit from capitalizing on high status community members to deliver anti-smoking messages through a variety of media.

There are a number of limitations to this research. With respect to Phase I, the large volume of data potentially available required a finely-developed document search and retrieval strategy. While decisions to reject or keep sampled documents were guided by the research protocol, it would be impossible to review all available documents in the TDO system. With respect to Phase II, we had a relatively small sample size and low participation rate which adversely affects the external validity of our results. Participants who volunteered for the study may differ in substantial ways from those who did not. Quite possibly, focus group participants held stronger anti-smoking views and these views may not be representative. All focus group participants were attending school full-time and the opinions of AA youth not attending school were not obtained. As smoking is related to school drop-out, this limits the transfer of these findings. With respect to qualitative methods, it is common to continue to collect data until a saturation point is reached. Though saturation within each focus groups was achieved, full theme saturation across groups may have been restricted by the number of participants studied. Finally, it is unclear why youth focus group participants in the Washington, DC metropolitan area would evidence relatively little awareness of the American Legacy Foundation's truth counter-marketing campaign when this area of the United States was rather heavily saturated with the campaign. One possibility is that our focus group participants were too young and cognitively immature to appreciate the campaign's complex messages. Prior work has shown this might be true [16].

Key phases in designing a tobacco counter-marketing campaign for AA youth in Washington, DC included reviewing tobacco industry documents, identifying common marketing themes and tactics, and providing examples of the marketing of cigarettes and other tobacco products to AAs and youth. These results are clear, pointing to the high degree of industry commitment to recruiting and retaining young AA smokers and their preference for mentholated brands. Other key phases included conducting qualitative research with AA youth, deepening the understanding of the role of smoking in their lives, and gauging the extent to which they may feel manipulated by the tobacco industry. The work suggests much room for improvement in AA youths' literacy about tobacco industry marketing, and the potential contribution of counter-marketing campaigns to control tobacco use in this population. More research in this area is needed to fully achieve this potential, and to do so in a culturally-competent manner.

In conclusion, this research indicates AA youth have long been a target of tobacco industry manipulation to entice them to smoke. Though explicit recognition of these tactics may be challenged among some young people, counter-marketing approaches clearly drawing attention to such tactics by informing young AAs about them hold promise as a means to reduce smoking.

## Authors' contributions

All authors read and approved the final manuscript.

## Availability & requirements




